# Using evaluability assessment to assess local community development health programmes: a Scottish case-study

**DOI:** 10.1186/s12874-017-0334-4

**Published:** 2017-04-21

**Authors:** Melissa Belford, Tony Robertson, Ruth Jepson

**Affiliations:** 1 Scottish Collaboration for Public Health Research and Policy (SCPHRP), University of Edinburgh, 20 West Richmond Street, Edinburgh, EH8 9DX UK; 2 Centre for Public Health and Population Health Research, Faculty of Health Sciences & Sport, University of Stirling, Room J04, Pathfoot Building, Stirling, FK9 4LA Scotland UK; 3 Lead for Evaluation Research, Scottish Collaboration for Public Health Research and Policy (SCPHRP), University of Edinburgh, 20 West Richmond Street, Edinburgh, EH8 9DX UK

## Abstract

**Background:**

Evaluation of the potential effectiveness of a programme’s objectives (health or otherwise) is important in demonstrating how programmes work. However, evaluations are expensive and can focus on unrealistic outcomes not grounded in strong theory, especially where there is pressure to show effectiveness. The aim of this research was to demonstrate that the evaluability assessment (a cost-effective pre-evaluation tool that primarily gives quick, constructive feedback) can be used to help develop programme and outcome objectives to improve programmes while they run and to assist in producing more effective evaluations. This was done using the example of a community development programme aiming to improve health and reduce health inequalities in its target population.

**Methods:**

The setting was Glasgow, Scotland, UK and focused on the Health Issues in the Community programme. Data were collected from documents and nine individual stakeholder interviews. Thematic analysis and a realist approach were used to analyse both datasets and, in conjunction with a workshop with stakeholders, produce a logic model of the programme theory and related evaluation options to explore further.

**Results:**

Five main themes emerged from the analysis: History; Framework; Structure and Delivery of the Course; Theory of Action; and Barriers to Delivery and Successful Outcomes. These themes aided in drafting the logic model which revealed they key programme activities (e.g. facilitating group learning) and 23 potential outcomes. The majority of these outcomes (16) were deemed to be short-term outcomes (more easily measured within the timeframe of an individual being involved in the programme) e.g. increased self-esteem or awareness of individual/community health. The remaining 6 outcomes were deemed longer-term and included outcomes such as increased social capital and individual mental health and wellbeing.

**Conclusions:**

We have shown that the evaluability assessment tool can be applied to the evaluation of community health programmes, providing short- and long-term outcomes that could be evaluated to demonstrate effectiveness and avoid unnecessary or poorly designed full-scale evaluations. This type of pre-evaluation method is already a useful resource for national policy evaluations, but could be a valuable evaluation tool for other regional or community health programmes.

## Background

Health inequalities are an on-going, world-wide issue [1]. Among the earliest British publications are the Black Report and the Acheson Report which detail that health inequalities are correlated with the social determinants of health such as age, gender, educational attainment, ethnicity, employment status, income level and geographic location [2–4]. Such inequalities include discrepancies in access and availability of health resources to groups with lower socioeconomic status (SES). Poor health outcomes that are related to lower SES include increased risk of death in young children, increased maternal mortality, chronic diseases, and decreased life expectancy [5]. Such consequences are seen in societies across the world, making health inequalities a substantive global public health issue. Assessments such as the Marmot report show that some inequalities are slowly improving over time thanks to national and local programmes and policies [6]. These changes bring about the need for evaluation as it is important to understand which policies and programmes work and in what contexts in order to be cost-effective and sustainable in terms of improving population health while reducing health inequalities.

A reduction in health inequalities is a long-term goal of many local and national governments and the most effective strategies (in terms of magnitude of effect) are likely population-level, ‘top-down’ interventions at the structural or regulatory level (e.g. taxes, trade policies etc.). However, it is important to take into account both short-term objectives and bottom-up approaches (community and local-driven needs and actions) as a way of complimenting top-down approaches to help create long-lasting and sustainable change and effective polices [7–9]. Such a community-based intervention from the UK that was developed with support from the public sector is Health Issues in the Community (HIIC), a community educational training programme based in Glasgow, Scotland and delivered throughout Scotland. This two-part training programme, with a focus on peer-led learning and community development, aims to increase community capacity and community participation, as well as to establish and consolidate community development approaches through confronting health inequalities [10]. The course is generally taken up by people within a local, geographic community looking to be equipped with the tools for developing community responses to health issues and to be more active citizens. The HIIC course is delivered by trained tutors and can be delivered as a nationally recognised accredited course. The programme uses a social model of health which recognises that an individual and their behaviour is affected by wider social determinants as well as an assets-based approach which looks at what participants can bring to the programme rather than focusing on victim-blaming [4, 11]. The long-history of HIIC hints at effectiveness in terms of health improvement, but attributing effectiveness to a single programme in health improvement and reducing health inequalities can be difficult, which establishes the need for better designed evaluations [12].

Health inequalities are complex issues that are hard to tackle and correspondingly, hard to evaluate [13]. While process evaluations have been conducted for community-based interventions, there is still a lack of routine evaluations done in terms of evaluating the health effects associated with such interventions [14, 15]. However, evaluations of programmes cannot be carried out without understanding the theory behind the programme and what outcomes are of greatest interest across stakeholder groups. The aim of this study was to facilitate the development of an evaluation of HIIC that would allow for feedback and measures in terms of effectiveness and sustainability of outcomes in terms of improving health and reducing health inequalities that could also be generalised to other programmes that are based on community development theory. Generalisability has become less evasive in qualitative research by using similar measures of validity across interventions [16]. Community development evaluations have focused on measuring cost-effectiveness and assessing design, but it is important to look at generalisability to examine if the desired outcomes are occurring and to what degree [17, 18]. In order to unpack such a complex intervention before evaluation, an evaluability assessment can be used. An evaluability assessment is a cost-effective pre-evaluation tool that primarily gives quick constructive feedback to the stakeholders, usually the administrators and funders, helping to develop programme objectives and translates research into practice by examining feasibility, acceptability and generalisability or transferability (if the intervention itself could be used for a particular population) of the intervention. This method of assessment has been around since the 1970s although has recently grown in popularity, especially in public health intervention [19].

There are six main steps to an evaluability assessment: 1) meeting with and getting involvement from the intended users of the evaluation; 2) clarifying the intended purpose of the assessment with these users; 3) exploring the programme reality with stakeholders; 4) reaching agreement on activities or goals highlighted; 5) exploring a range of alternative evaluation designs; 6) agreeing on evaluation priorities and intended uses of the information generated from the previous stages [19, 20]. We present our methods and findings from using this six-step model of evaluability assessment of the HIIC programme. We then discuss these findings to be generalisable to other community programmes, particularly those with an interest in improving the health of participants.

## Methods

### Setting

The study took place in Glasgow, Scotland, UK during the months of May to July 2015.

### Sample

The study sample was comprised of representative respondents from several key stakeholder groups involved in HIIC. There are several groups and organisations which each hold an essential stakeholder position in HIIC, making their opinions and experiences of the programme of interest to the present study. The key stakeholder groups consisted of:

1. Members of the Scottish Community Development Centre/Community Health Exchange (SCDC/CHEX), which is the organisation in charge of administering HIIC.
2. NHS Health Scotland (NHS HS, the national health improvement and health education provider), which is the funding body of HIIC.
3. HIIC tutors, who are the people who deliver the HIIC course.
4. Academics with a background in community development or assets-based approaches who are aware of the underlying theory, strengths and weaknesses behind such approaches as community-led health.

#### Stage 1.1: Documentary analysis

To start, the research team met the party commissioning the evaluability assessment, SCDC/CHEX, and was given relevant reports such as an Evidence of Impact Report [21] and a document giving background on the HIIC’s underlying approach entitled Community Led Health for All [22] as well as the HIIC pack which contained the materials a tutor would draw upon during the delivery of the course. Other relevant sources of data such as the HIIC website and case studies written by one of the HIIC tutors were also retrieved. Two different logic models were obtained to contribute to the identification of the preliminary programme theory and logic model. The first was a model drafted by a HIIC tutor representative of HIIC courses running from 2005–2008 in the North Lanarkshire region of Scotland. The second logic model was compiled by CHEX and was embedded in the Community Led Health for All Report [22]. Further documents on quantitative data such as number of attendees and tutors, as well as post-course survey summary data, were retrieved from the commissioner via e-mail.

#### Stage 1.2: Stakeholder interviews

Semi-structured interviews were conducted with nine relevant stakeholders. Eight of the interviews were face-to-face and one interview was conducted over the telephone, all of which were audio recorded using digital recorders. The interview guide was organised around participants’ views about the original aims, assumptions, outcomes, and effectiveness of HIIC. Depending on the participant’s profile, questions were adjusted accordingly, i.e. more theoretical questions were asked to academics and questions about internal workings of the programme were addressed to members of SCDC/CHEX. Consequently, due to the semi-structured nature of the interviews, the researcher did not strictly adhere to the topic guide, but rather kept the conversation within the broader topics addressed i.e. origins, outcomes, assumptions, and effectiveness.

#### Stage 1.3: Development of initial logic model

The last component of Stage 1 was drafting a logic model depicting the current programme theory of HIIC which addressed how HIIC could be evaluated in the future and to articulate the underlying programme theory. The programme theory was identified from an analysis of: 1) the training manual and related documents, and 2) the semi-structured interviews with stakeholders. Programme theory refers to the development of linking programme inputs and activities to the intended or observed outcomes and then using this model to inform evaluation [23]. This was done primarily by drawing from the thematic analysis and realist evaluation to draw themes from the data i.e., the coding and interviews. The documentary analysis was also used to compare the past programme theory to the current findings.

#### Stage 2.1: Workshop

For Stage 2, an interactive workshop was held with five of the participants who had been interviewed earlier, representing all stakeholder groups. During the workshop, stakeholders were presented with the logic model (in large poster form), consisting of short-term and long-term outcomes. The research team explained the model and asked for feedback as to whether any aspect of the programme theory was missing or if anything that was there didn’t belong.

#### Stage 2.2: Logic model refinement

**Fig. 1:**
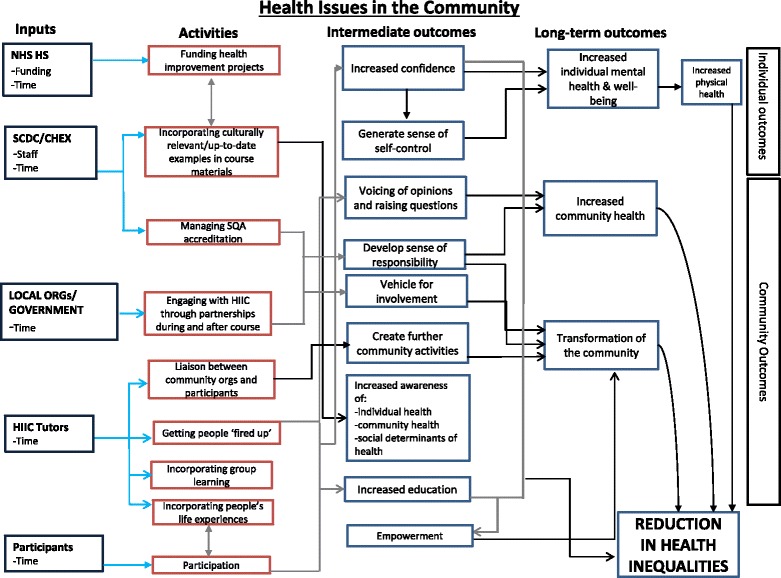
Logic Model of the casual pathways that, according to stakeholders, explain how HIIC works

Following the workshop, the stakeholders’ feedback was taken into account and the logic model was revised. The revised model can be found in the results section (Fig. 1).

#### Stage 3: Report

During the initial consultations with SCDC/CHEX with regards the purpose of te evaluability assessment, various alternative evaluation scenarios were discussed, ranging from no resources (time and/or staff) to unlimited resources. Based on these discussions, the research team was asked to focus primarily on recommendations where only limited, short-term resources would be available, but to also include supplementary plans in case additional resources became available.

### Analysis

The research team used an iterative process of reviewing the documents and developing a current programme theory throughout the research process. Thematic analysis was applied to the documentary and transcribed interview data [24, 25]. Researcher MB then grouped relevant codes together in order to identify further patterns within each theme. Through discussion with the research team and revisiting the transcripts, a final set of themes and sub-themes of the codes were agreed upon.

Mapping and interpretation then took place whereby all relevant data items were matched to codes and the implicit programme theory was specified resulting in the production of the initial logic model. This data encompassed materials in the documentary analysis, primarily the past logic models and the excerpts from the interviews. The data were reviewed by the whole research team and after several discussions, consensus was reached as to the most representative interpretation of the data. The resulting programme theory was translated visually in a logic model that not only maps out these points, but describes the pathways between inputs and activities as well as relationships within activities. Furthermore, the logic model goes on to depict the pathways between activities and outcomes as well as the relationship within outcomes. The representation of these complex relationships is one of the benefits of creating a logic model because it gives insight into the outcomes and pathways of interest relevant to evaluation.

At the workshop, the stakeholders were asked to prioritise both the short- and long-term outcomes in order of need for evaluation. This included adding activities and outcomes as well as removing the outcomes that stakeholders did not view as relevant to the HIIC programme theory. Based on the stakeholder feedback and the collective data, four evaluation options were developed and these options were presented to SCDC/CHEX in a written report summarising the evaluability assessment [26].

## Results

### Stage 1 results: Themes

**Fig. 2:**
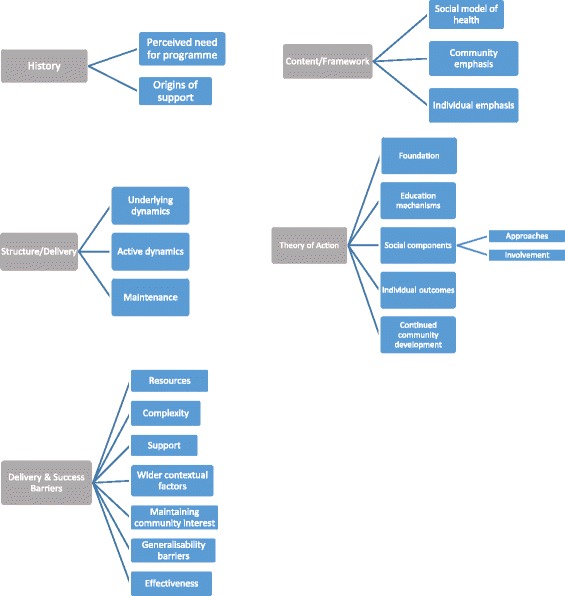
Main themes and sub-themes drawn from the stakeholder interviews

The analysis of the documentary review and the nine stakeholder interviews revealed several emerging patterns pertaining to the HIIC course that were organised into five broad themes and several further sub-themes (Fig. 2). These established themes reveal the predominant components that feed into the HIIC programme and accordingly, serve as the basis of the logic model.

#### Theme 1: History

Participants identified several components of HIIC’s origins from which two main sub-themes emerged with the first being the Perceived Need for the Programme (given the scale of health inequalities in Scotland and a history of community-development approaches) and the Origins of Support (from communities, the non-profit sector and the public sector).

#### Theme 2: Framework

The next theme encompassed the course itself which details what principles the course draws upon and three sub-themes were extracted with the first being the utilisation of the ‘Social Model of Health’ as a way to emphasise the wider determinants of health and to not solely focus on the individual level. Topics that arose from this sub-theme include addressing social determinants and inequalities and the use of culturally relevant examples in the content of the course.

#### Theme 3: Structure and delivery

The underlying dynamics of the course were discussed such as the course being informed by relevant policy and the fact it has standardised elements that are delivered irrespective of individual tutors or participant groups, while still retaining a flexible and adaptable format to match the needs of the tutor and participants. This serves to justify that the structure takes updated and new policies into account, demonstrating an iterative and reflective process. The delivery is accessible since it can be tailored to a particular interest group or other community setting by having materials accessible and that these materials are only changed when they are no longer relevant or there is an update or more relevant resource available.

#### Theme 4: Theory of action

This theme encompasses the different mechanisms that are believed to produce, or at least be associated with, programme outcomes.

- Foundation
*Foundation* entails the inputs needed to keep the course running: structural support, resources, capacity, promotion and accountability. These components were identified by stakeholders as the essential inputs and resources needed to just keep the programme operational that can be affected over time due to a lack of resources.
- Educational Mechanisms
*Educational Mechanisms* includes processes of increased awareness, increased education, knowledge, interactive or positive learning, group learning, critical thinking, and voicing opinions or questioning assumptions and information presented. This establishes that the learning component does not end with reading and discussing the course materials, but that gaining skills or capacity such as critical thinking and voicing opinions are part of a groundwork that allows participants to continue and extend learning into wider areas of their lives.
- Social Components
*Social Components* that play a role in the outcomes of the programme appeared in nearly every interview. Further aspects within this sub-theme that emanated includes Social Approaches: the need for upstream approaches and social justice, targeting the appropriate groups and transforming communities; and Social Involvement: engaging with frontline staff, partnerships with local organisations, networks, /common problems, and incorporating people’s lived experiences. This shows that the programme is not aiming to just improve individual health, but that it is essential to integrate social approaches and to stimulate involvement at different levels in the individual as well as the community. Social Approaches can be seen as a theoretical component and Social Involvement as the implementation of such theory in practice, with both necessary to achieve outcomes.
- Continued Community Development
*Continued Community Development* has some overlap with other components of the theme and covers a large proportion of the mechanisms including: getting people ‘fired up’, voicing opinions and questioning, people’s engagement, increased confidence, empowerment, sense of responsibility, creating further community opportunities, identifying opportunities, precursors to activism, vehicles for involvement, and networks, social cohesion and social capital. These community development outcomes show a journey of sorts that participants go on in terms of getting interested, gaining capacity and then engaging in communities. The findings of this sub-theme show that community development does not end after an intervention and that networks and opportunities need to be established and maintained in order to have lasting community outcomes.

#### Theme 5: Barriers to delivery and successful outcomes

This final theme addresses the Barriers to Delivery and Successful Outcomes that were discussed in the stakeholder interviews. This theme had a large variability in opinions and was further separated into seven sub-themes:

- Resources
  Several stakeholders discussed that there are certain components of the course that may be lacking and, thus, HIIC is not operating at its highest potential. These resources are ones external to the course structure and delivery and can be categorised as operational and administrative resources. Such inputs include a lack of regulation of the delivery and the results, lack of available data for evaluation, limited reach, and issues with time and commitment to the course from all the stakeholders.
- Complexity
  This sub-theme details the intricacies of the programme. The fact that HIIC is a complex intervention came across in the interviews as well as other complex issues including that the programme doesn’t address structural inequalities, there is no ‘cherry picking’ in the course, the language of community development and health can be confusing, there is a blur between outcomes and methods, and there is the complexity of social determinants in people’s lives.
- Support
  Lack of support was evident on several levels including achieving further involvement and support from the community, local organisations and government. This barrier can be linked to narrow and limited support, organisational restructuring, the hierarchy of stakeholders, different stakeholder expectations, and misunderstood aims.
- Wider Contextual Factors
  With any intervention, one needs to take into account that it is not isolated, meaning that wider systems will affect the community and people’s lives. Context needs to be considered when looking at effectiveness and evidence of impact because there may be external factors affecting impact. This barrier is illustrated by components of locating people’s behaviour in a wider context, the economic climate, and the programme’s level of political recognition (or lack thereof).
- Maintaining Community Interest
  Since HIIC utilises a community-led approach, it is essential that the participants (the community members themselves) are actively being listened to and that their needs are what lead the course. Often interventions can be prescriptive, and it is important to keep the community in charge.
- Generalisability Barriers
  A key component that evaluation assesses is how results in one community can be compared to a wider population. If the programme is intended as a national intervention, the results need to be generalisable to the whole of the country (in this case Scotland) and transferable to a variety of communities. Specific generalisability issues with HIIC include that there is no set baseline for participants; they come from various educational backgrounds; there can individual differences in outcomes and levels of engagement; and different needs for different community groups.
- Effectiveness
  In general, every intervention is out to prove effectiveness which can be difficult due to complexity of overlapping causal pathways. In terms of HIIC, stakeholders discussed that it is hard to prove that the programme itself is causing the change (‘attribution’) and that the funding is justifiable (‘cost effectiveness’).

### Stage 2 results: Workshop and logic model refinement

By using the results of Stage 1 as a foundation, the development of the logic model revealed several different activities and pathways to the outcomes discussed by the stakeholders. The logic model is one representation of how a number of key stakeholders view the programme theory, but may not represent the views of everyone. The logic model is an evolving data instrument as a programme develops and more people become involved. Due to the ethical risk of making participants identifiable, we cannot divulge the number of stakeholders in each group, however, all key stakeholders were invited and participated and the workshop process allowed them to review what had been developed based on the interviews. From the analysis, five main clusters of inputs were identified which reflect the theory of change component of the programme theory, what mechanisms and processes in the context of the programme cause the change to occur. The inputs identified corresponded with relevant stakeholders and the logic model displays what activities and outcomes they contribute to. For example, the inputs of funding and time by NHS HS lead to the activity of funding health improvement projects which then leads to the outcome of increased confidence as well as the other intermediate outcomes that result in empowerment. This casual path continues to lead to the long-term outcome of increased individual mental health and well-being and increased physical health which results in the impact of a reduction in health inequalities (given this programme is targeted in more deprived communities).

Stakeholder feedback on the model was achieved through the interactive workshop. This workshop revealed that the inputs from the HIIC tutors and participants were believed to be the essential components for the success of HIIC (obviously supported by funding and time from the NHS and SCDC/CHEX). These are the two stakeholder groups that are physically present throughout an active HIIC course so their inputs such as time and ‘bringing themselves’ are necessary to produce the intermediate outcomes that lead to long-term outcomes.

Through the discussion, it was revealed that there were clusters of outcomes that were most important to the stakeholders. These outcomes are described in detail in Table 1. In summary, for the short- to medium-term outcomes the clusters that emerged were: increased confidence; empowerment; self-efficacy; understanding attitudes; voicing of opinions and raising questions; and increased awareness and learning and development. The three long-term outcomes that the stakeholders prioritised were: increased individual mental health and wellbeing; enhanced social capital; and increased health of communities.

**Table 1:** Prioritised outcomes

Short-term outcomes
*1. Increased confidence, Empowerment, Self-efficacy* This cluster was prioritised as the most important set of intermediate outcomes to assess. The outcomes were perceived by stakeholders as a group of individual based outcomes that establish the foundation for other community and long-term outcomes to occur. Increased confidence was perceived by stakeholders as one of the key outcomes that, coupled with an increase in self-efficacy (one’s belief in their ability to achieve an outcome), leads to participants feeling empowered at an individual level. This individual empowerment can then result in improved individual mental health and wellbeing. This can also lead to a collective community empowerment that has been linked to incorporating group dynamics and utilising interactive learning [36].
*2. Understanding attitudes, Voicing of opinions and raising questions* This set of outcomes emphasises the ability and willingness of participants to critically think and vocalise their thoughts. These outcomes are associated with a higher retention of knowledge and an increased uptake of critical thinking skills [37].
*3. Increased awareness, Increased learning and development* This last pair of outcomes incorporates learning about health with an increased awareness of health, including awareness of individual health, community health and the social determinants of health. Consequently, this increase in awareness mirrors an increase in learning and development [38].
Long-term outcomes
*1. Increased individual mental health and wellbeing* Measuring the change in mental health and wellbeing was important to the stakeholders because it has been shown that an improvement in health can have positive effects and elicits other changes. It can also be a precursor to improved physical health which could contribute, with improved mental health, to the reduction of health inequalities [39].
*2. Enhanced social capital* Social capital refers to social networks and the resources that are available through these links [40]. Gaining social capital is associated with improved mental health and wellbeing and increased interaction in the community showing a sense of social cohesion (the willingness of groups/communities/societies to interact and cooperate with each other) among the individuals and the community agencies and other members. One example of enhanced social capital in HIIC is at the end of Part 1 when participants work together to research an issue in their community. In order to engage fully with the issue, participants often contact other members of the community for example, to try to keep a local park free of safety hazards such as broken glass and syringes, they may contact police to enquire if the area is patrolled at night, which then allows for the possibility for the participants to invite the community members to their presentation thus establishing social cohesion through the outreach.
*3. Increased health of communities* This outcome reflects the community-wide changes that come from the course with potential outputs being increased volunteering, further activity in community projects, and increased physical health. Community health can be improved by engaging participants in an intervention and using the data to shape plans for further community goals which reflects HIIC’s community-led approach [41].

This feedback on the outcomes allowed for the model to be revised, reflecting the goals and priorities of the collective cohort. At the end of the workshop, three key evaluation questions were formed that were applicable to each of the outcomes of interest for the short-, medium- and long-term outcomes:

1. Has HIIC improved [INSERT OUTCOME HERE] (e.g. ‘increased confidence’)?
2. If yes, how has HIIC improved this outcome? If not, why not?
3. What has followed from this outcome?

These key evaluation questions have been taken into consideration while developing the evaluation options.

### Stage 3 results: Report on evaluation options

Based on the results of Stages 1 and 2, a series of evaluation strategies in the form of recommendations can be made. The evaluation options specific to HIIC can be found elsewhere [26]. It is important to provide a range of options to allow programmes to decide which options best match their resources and priorities (for example, collecting cross-sectional or retrospective data versus longitudinal data).

## Discussion

This study undertook the principles of an evaluability assessment in order to understand how a community-based programme aiming to improve health and reduce health inequalities worked, how relevant professionals believe it works and how it could be evaluated in the future. This was achieved by developing a logic model; determining theoretical links between the programme and health and social improvement; and determining the potential the programme has to affect a range of outcomes that might impact health inequalities. The findings show that community-based programmes with well-developed support from stakeholders (such as HIIC) experience great uncertainty when it comes to specific issues such as attribution, sustainability and transferability, as well as some stakeholder expectations. These findings are key to the planning and delivery of future evaluations, as well as the current planning and delivery of this programme and other similar community-based health programmes.

### Findings in relation to other studies

While community development programmes have been encouraged and supported to carry out evaluations of their programmes and activities, [27, 28] there remains an absence of robust evaluations, [29] especially in programmes with health as their main focus. Typically, these programmes are not much further ahead of HIIC in terms of evaluation - they have often had internal evaluations conducted by their funders or other community organisations that look at the intermediate outcomes of the programme, but say little about long-term impact i.e. the reduction in health inequalities [30]. This study utilised the evaluability assessment methodology to not only provide a logic model and underlying programme theory for HIIC to use to evaluate their programme, but it can also be used in the wider community health development arena where there are a vast amount of community-based interventions, yet robust evaluations are scarce [31]. One exception is the evaluation conducted for Robert Wood Johnson Foundation’s Urban Health Initiative, which used a similar method to the present evaluability assessment to evaluate a theory of change for a community health initiative [32]. Although similar, this evaluation relied more on quantitative data for outcome evaluation. While the foundations of such evaluations are similar to the ones carried out in the HIIC evaluability assessment, the present programme differs given a lack of existing data and due to the need for more of a qualitative approach to identify the outcomes of interest that can be measured in future evaluations.

Due to the variety of definitions in terminology as well as the differences in what outcomes are measured and the population in question, reviews of such interventions assessing community development and health outcomes are unable to synthesise data and draw robust conclusions [24, 29, 33]. This study shows that there are limitations to what can generalised from an individual study and that evaluations should try to look at outcomes that are generalisable across several community development studies [34]. Evaluability assessments have been utilised in policy work and have revealed results and ideas that wouldn’t have been thought of otherwise. There is little application to community programmes which could be beneficial, as this research found, in terms of identifying priorities and evaluating outcomes.

### Relevance of results

The logic model produced by the current study elaborates on past logic model attempts for HIIC. For example, the more recent Learning, Evaluation and Planning (LEAP) model shows that HIIC is situated within wider systems, but does not detail the programme theory of the course [22]. The logic model completed in 2005 by a HIIC tutor follows the community-led structure, but is community specific: it is more of an explanation of the course after it has occurred through the community development principles rather than an organic formation of a programme theory. What the current logic model adds is a more academic perspective to explaining how HIIC works through causal pathways according to the involved stakeholders (Fig. 1). This is valuable in challenging some of the underlying assumptions and external contexts, such as the assumption of a particular participant’s ‘profile’ (their age, gender, occupational status, previous educational experiences and qualifications and mental health). These assumptions and external contexts may be as important, if not more important, in influencing outcomes compared to the intervention itself. We have presented a unique application of an evaluability assessment to a community programme, allowing for insights that standard evaluations often disregard. This helps show the potential value that widespread utilisation of evaluability assessments could have for community programmes, especially those focusing on health outcomes and changes.

One difficulty in an evaluability assessment is navigating power relationships between stakeholders. This is confirmed by data from the semi-structured interviews, which was teased out in the ‘Support’ sub-theme under Theme 5 ‘Barriers to Delivery and Successful Outcomes’, that reveal that stakeholders felt a presence of a hierarchy among stakeholders and that often the various stakeholders have different agendas and thus different priorities. This often resulted in a lack of maintenance in the implementation, delivery and follow-up of the programme. It is important to note that after interventions, community development is not over. Continued engagement and involvement are essential components of community development [35]. After the development occurs, continued participation, as well as maintenance, need to be considered so that the results will be sustained. This is something that needs to be applied across community development programmes in order to promote sustainability.

### Key themes

Several themes emerged in the individual interviews, whereas other themes were teased out through the workshop and logic model refinement. Themes that were at the forefront of most interviews were the importance for continued funding support by NHS HS for validity and the resources to keep the programme running, as well as a need for stakeholders to attempt to reach agreement on the core issues. There was also consensus that the peer-led structure of the course is an essential component and that the flexibility of the course allows HIIC tutors to customise the course to their community. Themes that were drawn out further in the analysis centred on the broader long-term outcomes. Through discussion at the workshop, the stakeholders were able to agree that although HIIC aims to reduce health inequalities, effectiveness would typically be visible by looking at more concentrated long-term goals. This point did not come up in the individual interviews and was brought to light when the research team went through the preliminary logic model, which had ‘Reducing Health Inequalities as the long-term goal. The combination of the discussion and the visual representation of the programme structure and outcomes via the logic model allowed the stakeholders to reach a consensus that a future evaluation of improved mental health and well-being, increased social capital, and increased health of the community are long-term outcomes that move towards the direction of reducing health inequalities.

Although this evaluability assessment of qualitative data required a lengthy iterative process of interpreting data and themes, it is important to note that this process is not always required at such length for all evaluability assessments. However we felt it necessary because SCDC/CHEX felt they ‘knew’ that participants benefit from HIIC, but they came into this project with limited understanding of what components of the programme produce the beneficial outcomes and how best to measure these. This iterative process was used to generate a comprehensive understanding of the programme from the materials and the stakeholders involved.

### Strengths

The major strength of this study was the novel use of the evaluability assessment method in the context of community-based programmes and interventions. This method can only be truly effective if there is a willingness of participants to take part. This willingness was first apparent by the high level of responses across the stakeholders. The evaluability assessment provided insight into the aims and desired outcomes of the HIIC programme (and as a model for other programmes), but it is necessary to note that this is just one step in the evaluation process. A full evaluation entails more time and resources as well as cooperation and agreement on goals by the stakeholders. It needs to build upon the evaluability assessment and continue the iterative process of consulting stakeholders and refining the programme theory. Until a full evaluation is performed there is only an insight into parts of HIIC, and a full picture is not accessible. However, such evaluability assessments can identify key areas of interest and knowledge gaps before full-scale evaluations are carried out.

The logic model has allowed for the identification of what the stakeholders believe to be the underlying programme theory of HIIC which will be useful in the future research to test these mechanisms, and also in the continued engagement and understanding of different stakeholders. This was already demonstrated during the interactive workshop as stakeholders exchanged and debated preconceptions and perceptions of HIIC in order to better understand how it works in theory and reality, as well as discussing what should be prioritised in any future evaluations. This is a key example of how the evaluability assessment methodology can go beyond what is available through qualitative interviews alone, with the importance of the facilitated workshop central to allowing stakeholders to see, reflect on and discuss the logic model. Without this step, many of the outcomes would not have emerged as clearly.

### Limitations

The main limitation of this evaluability assessment was that HIIC participants were not consulted as a stakeholder group due to the complexity of ethical permissions. It is important to acknowledge that the participants’ involvement in the discussion may have added to the logic model. With this in mind, the researchers recommended the inclusion of HIIC participants in the proposed evaluation plan. An evaluability assessment does have several linear steps, however some steps need to be revisited and such a method can be seen as an iterative process. Even once an evaluability assessment has been carried out, changes to the programme design or delivery could be incorporated into iterations of the logic model and evaluation options. Other research design limitations are that only one programme was assessed which makes it more difficult to generalise the results to other programmes. This was mitigated by the fact that community development programmes are usually based on similar principles. Additionally since this was an evaluability assessment, a full evaluation was not carried out so further evaluation needs to occur before clear statements can be made of the efficacy of HIIC.

Although the logic model is useful, it is not without its own set of limitations. The current logic model depicts how the stakeholders view how the programme works, so it is necessary to further test out the programme theory since some of these pathways may just be based on assumptions. The underlying programme theory seems plausible yet ambitious as a whole, but the actual processes such as how increased individual and mental health can lead to increased community health and transformation of the community needed to be tested and/or supported further by both theory and evidence.

Logic models are often based on a series of assumptions that may or may not be plausible. One of the reasons why plausibility is more likely in this study is that the logic model, and associated assumptions, are developed with the people who developed and deliver the intervention. It would have been less plausible, for example, if it had been developed by an evaluator without such input or based purely on theory. One way to further test the plausibility is to undertake an evaluation which tests the programme theory and the underlying assumptions. So qualitative or quantitative data can be collected for some of the intermediate outcomes to see whether the theory outlined in the logic model works as intended. Logic models often need revisiting and revising in the light of new contradictory data, but can be very useful in determining how we get from activity (a) to outcome (c) by pathway (and intermediate outcome) (b). So at this stage they are hypothetical to some extent and can only be ‘proved’ or disproved’ by collecting data. The purpose of an evaluability assessment is partly to generate ideas for testing plausibility. We would normally recommend a theory-based evaluation if the plausibility of the theory was in doubt.

### Future work

This study demonstrates that outcomes are complex, interrelated and often interact and feedback with one another, making it challenging to isolate in such a complex intervention. This makes it difficult to explicitly prove causality of particular outcomes of interest. A further evaluation looking at the stakeholders’ prioritised outcomes of interest would provide the opportunity to further understand variables that contribute to specific outcomes. This would then allow researchers to isolate what variables to look at and a greater opportunity to prove how certain pathways help to reduce health inequalities.

Overall the evaluability assessment was an additional component to the development of a strong, robust evaluation plan that could link the activities of the organisations with the outcomes that need to be addressed, especially since HIIC has limited resources for evaluation. This aligns with most other community development programmes which is why the idea of assessing impacts should be done in an evaluation that encompasses several studies or starting with a single study that could represent a variety of community development studies [29].

The findings from this evaluability assessment and the potential findings from a later evaluation are generalisable to other studies on several levels: HIIC utilises current national policy and health improvement reforms in order to guide the course; the underlying principles of community development are present in the programme theory such as participation, engagement and empowerment; the intermediate outcomes are based on individual and community health improvement like other studies; and the long-term impact to sustain the outcomes that have transformed the community and to ultimately reduce health inequalities. These goals are shared among community development interventions so evaluating HIIC will give insight into other programmes that can be compared and contrasted to.

## Conclusions

This study used evaluability assessment to explore the activities and outcomes of a programme that uses community development approaches to improve the health and wellbeing of deprived communities. Although there may be differences between programmes and it is still not certain how generalisable these results are, the evaluability assessment method could be employed in other, well-established programmes with a mix of stakeholders to help produce new thinking and the possibility of more focused and effective evaluations. The next step would be to take the findings of this evaluability assessment forward to an evaluation and continue to develop the logic model with the input of participants.

## Abbreviations

CHEX: Community Health Exchange
HIIC: Health Issues in the Community
LEAP: Learning, Evaluation and Planning
NHS HS: National Health Service Health Scotland
RCT: Randomised control trials
SCDC: Scottish Community Development Centre
SES: Socioeconomic status
